# Supplementary material for: The cost and cost-effectiveness of novel tuberculosis vaccines in low- and middle-income countries: A modeling study
Source: PLoS Med. 2023 Jan 24;20(1):e1004155. doi: 10.1371/journal.pmed.1004155 (PMC9873163; doi:10.1371/journal.pmed.1004155)
Supplement: Attachment — Submitted filename: Response_to_Review_v1.docx [file pmed.1004155.s002.docx]

**Itemized Response for PMEDICINE-D-22-01725-R1**

This document describes clarifications and edits made in response to editor and review comments provided on PMEDICINE-D-22-01725-R1 (“*The cost and cost-effectiveness of novel tuberculosis vaccines in low- and middle-income countries: a modelling study*”). In addition to these edits, we have also updated the results shown in the paper to correct for errors that were discovered in the programming code for this analysis. These edits change the numerical results of the analysis (9–16% increase in global-level ICERs) but not the qualitative conclusions. We apologize for these errors.

Comments from the Academic Editor:

I am in agreement with all of Reviewer #2’s comments and suggestions. They should provide more detail about what they did even if they do not reproduce the whole epi model. It is not reasonable to expect people to have to read their other papers to understand this one.

*Authors’ response:*

*Thank you for this suggestion, we have added additional information regarding the epidemiological model. These edits include the tuberculosis natural history structure, HIV and ART structure, calibration methodology, vaccine profile, vaccine delivery scenarios and coverage targets, and country-specific introduction year methodology. We provide a detailed description of each of these edits in response to Reviewer #2’s comments.*

I did have a couple of other concerns. The hypothetical vaccine they model has attributes that are very similar to BCG which is now widely used in almost all the countries covered. Specifically, vaccine efficacy in newborns is around 72% in neonates and 47% in adolescents and adults and the durability is around 10 years although this latter is controversial. The Clark model acknowledges high BCG coverage among neonates in most high burden countries but does not specifically integrate that into the model. They note that they envision the new vaccine would be in addition to BCG at birth but don’t mention whether they consider BCG revaccination.

Since the proposed efficacy of the new hypothetical vaccine in adolescents/adults is 50%, very similar to the efficacy of BCG in the Nemes South Africa clinical trial, the question arises of why not just use BCG at a tiny fraction of the cost. Presumably, the answer to that is that BCG revaccination efficacy has been highly heterogeneous in different areas – but that should serve as a warning that other TB vaccines may behave similarly depending on their biologic mechanism. It is also quite possible that children who "fail" BCG (ie. those who stand to benefit from another vaccine) are precisely those who might fail the new vaccine.

*Authors’ response:*

*Thank you for your questions regarding the vaccine profile assumed in our vaccine delivery scenarios. The specifications of both the adolescent/adult and infant vaccine were based on the WHO Preferred Product Characteristics (PPCs) for New TB Vaccines. For a novel adolescent/adult vaccine, it is recommended that the new vaccine prevents active pulmonary TB disease with a minimum efficacy of 50% and be efficacious in individuals with and without evidence of* Mtb *infection at the time of vaccination.*

*The vaccine characteristics described in the Nemes trial evaluating BCG revaccination are different from the characteristics recommended for an adolescent/adult vaccine in the WHO PPCs. BCG revaccination in the Nemes trial was administered to uninfected individuals and the outcome measured was prevention of sustained infection.*

*Therefore, for the adolescent/adult vaccine in our scenarios, we introduced a prevention of disease (POD) vaccine with 50% efficacy that would be efficacious if administered to both uninfected and infected individuals to align with the characteristics from the WHO PPCs. We assumed that the vaccine would have no impact on prevention of infection.*

*To clarify this, we have included more details in the manuscript and the appendix on the vaccine profile and delivery scenarios that describe the vaccine scenarios introduced in Clark et al, as described below. We have also edited the limitations section to note that this other vaccine type was not included in the analysis.*

**Methods, Pages 5, Lines 37–42:**

Compared to this baseline, we evaluated two different vaccine product profiles: an infant ‘pre-infection’ prevention of disease vaccine (i.e., efficacious for individuals uninfected at time of vaccination) with 80% efficacy targeting neonates, and an adolescent/adult ‘pre- and post-infection’ prevention of disease vaccine (i.e., efficacious in all individuals aside from those with active TB disease at time of vaccination) with 50% efficacy, based on WHO Preferred Product Characteristics for New Tuberculosis Vaccines.

**Discussion, Pages 18–19, Lines 332–336:**

We also restricted the analysis to focus on vaccine products that would prevent development of TB disease, and did not examine the possible impact of vaccine products that would prevent infection, such as recombinant BCG vaccines [1]. If successfully developed, such vaccines could provide another effective tool for accelerating TB control.

[1] Cotton MF, Madhi SA, Luabeya AK, Tameris M, Hesseling AC, Shenje J, et al. Safety and immunogenicity of VPM1002 versus BCG in South African newborn babies: a randomised, phase 2 non-inferiority double-blind controlled trial. Lancet Infect Dis. 2022;22(10):1472-83.

**Appendix S1, Pages 8–9, Lines 81–98:**

**S1.4. Vaccine profile**

The vaccine profile for an adult/adolescent vaccine and infant vaccine were based on the WHO Preferred Product Characteristics for New Tuberculosis vaccines [1], and are outlined in Table S3.1 below.

**Table S3.1.**	**WHO Preferred Product Characteristics for New Tuberculosis Vaccines.**

| **Vaccine** | **Host infection status at time of vaccination required for efficacy** | **Effect type** | **Vaccine efficacy** | **Duration of protection** |
| --- | --- | --- | --- | --- |
| Adolescent / Adult | Pre- and post-infection | Prevention of disease | 50% | Lifelong |
|  |  |  |  | 10 years |
| Infant | Pre-infection | Prevention of disease | 80% | Lifelong |
|  |  |  |  | 10 years |

Vaccine efficacy was assumed to be the same in both PLHIV and HIV-naïve recipients in countries incorporating the HIV structure, and in both younger age groups and older adults. The vaccine was assumed to have the same impact on preventing drug-susceptible and drug-resistant tuberculosis as specified in the WHO PPCs [1]. As we were modelling a prevention of disease vaccine, there was no direct impact on *Mtb* transmission or the force of infection.

We assumed duration of protection was 10 years on average, in addition to a sensitivity analysis with lifelong duration of protection. The shape of waning immunity was modelled as an exponential distribution, based on similar shapes for waning vaccine immunity of BCG [2] and other vaccines [3, 4].

[1] World Health Organization. WHO Preferred Product Characteristics for New Tuberculosis Vaccines. Geneva: World Health Organization. Licence: CC BY-NC-SA 3.0 IGO. 13 July 2018. https://www.who.int/publications/i/item/WHO-IVB-18.06 (accessed 18 October 2021).
[2] Abubakar I, Pimpin L, Ariti C, Beynon R, Mangtani P, Sterne JA, et al. Systematic review and meta-analysis of the current evidence on the duration of protection by bacillus Calmette-Guérin vaccination against tuberculosis. Health Technol Assess. 2013;17(37):1-372, v-vi.
[3] Blackwood JC, Cummings DA, Broutin H, Iamsirithaworn S, Rohani P. Deciphering the impacts of vaccination and immunity on pertussis epidemiology in Thailand. Proc Natl Acad Sci U S A. 2013;110(23):9595-600.
[4] Lewnard JA, Grad YH. Vaccine waning and mumps re-emergence in the United States. Sci Transl Med. 2018;10(433).

Another minor procedural point. The authors note that the funder (WHO) had no role in the study but at least one of the authors, Matteo Zignol, is from WHO.

*Authors’ response:*

*We have edited the financial disclosure section to reflect this involvement. Although this section is removed from the main manuscript per the editor’s request (8) below, the accompanying material including this information will read:*

“This study was funded by the World Health Organization (2020/985800-0). Members of the funder participated as authors on the study. All authors had the opportunity to access and verify the data, and all authors were responsible for the decision to submit the manuscript for publication.”

Requests from the editors:

1) Please confirm whether the underlying model has been submitted for publication elsewhere. Ideally, this model needs to be peer reviewed and published.

*Authors’ response:*

*Thank you for this question. We can confirm that the underlying epidemiological model has been submitted for publication at The Lancet Global Health and is currently Under Review. The paper and supplementary material are available as a pre-print at* [*https://www.medrxiv.org/content/10.1101/2022.04.16.22273762v1*](https://www.medrxiv.org/content/10.1101/2022.04.16.22273762v1)*.*

*We have added additional information on the underlying epidemiological model and vaccine delivery scenarios to the appendix (Appendix S1, pages 1–14).*

2) Abstract:
   a. Under methods, and findings, please summarize the data sources for key estimates

*Authors’ response:*

*We have added a list of some of the key data sources used in the methods and findings section of the Abstract. Additional details are included in the Methods section of the manuscript.*

**Abstract, Methods and Findings, Page 2:**

Key economic inputs were derived from the Global Health Cost Consortium, WHO patient cost surveys, and the published literature.

b. Please ensure that all numbers presented in the abstract are present and identical to numbers presented in the main manuscript text.

*Authors’ response:*

*We have reviewed the manuscript to ensure that all numbers in the abstract match the numbers presented in the main manuscript.*

c. In the last sentence of the Abstract Methods and Findings section, please describe the main limitation(s) of the study's methodology.

*Authors’ response:*

*We have added the following sentence to the end of the Abstract Methods and Findings to reflect the main limitations of this study.*

**Abstract, Methods and Findings, Page 2:**

Limited data availability required assumptions and extrapolations that may omit important country-level heterogeneity in epidemiology and costs.

3) At this stage, we ask that you include a short, non-technical Author Summary of your research to make findings accessible to a wide audience that includes both scientists and non-scientists. The Author Summary should immediately follow the Abstract in your revised manuscript. This text is subject to editorial change and should be distinct from the scientific abstract. Please see our author guidelines for more information: https://journals.plos.org/plosmedicine/s/revising-your-manuscript#loc-author-summary

*Authors’ response:*

*We have added the requested Author Summary following the Abstract in the manuscript:*

**Author Summary, Page 3:**

**Author Summary**

Why was this study done?

- Previous studies have highlighted the economic impact of tuberculosis (TB) disease and mortality, and the potential economic impact that novel TB vaccines could have on reducing this burden in specific low- and middle-income countries (LMICs).
- The cost and cost-effectiveness of novel tuberculosis vaccines, which depend on vaccine price and delivery strategy that may vary by country, are needed by vaccine developers, manufacturers, and potential purchasers to guide investment decisions.
- No modelling studies have estimated the cost and cost-effectiveness of novel TB vaccine products with country-specific assumptions for medical and non-medical costs, indirect costs, vaccine delivery costs, and delivery strategies across a wide range of LMICs.

What did the researchers do and find?

- We estimated the costs, cost-effectiveness, and incremental net monetary benefit of TB vaccine introduction from both the health system and societal perspective, in order to inform global-level decision-making for novel TB vaccine investment and introduction.
- Using mathematical and economic models, we assessed scenarios for the introduction of novel TB vaccines with a wide range of characteristics and a diverse set of health and economic outcomes, including country-specific introduction years from 2028–2047.
- Our analysis projected that an effective new TB vaccine could offer large potential health and economic benefits over 2028–2050. From a societal perspective, vaccination was projected to be cost-effective in 74 LMICs compared to a 1x per-capita gross domestic product threshold.
- When considering the monetized value of health gains, we estimated that introduction of an adolescent/adult TB vaccine could produce $283–474 billion in health and economic benefits by 2050, with greater benefits in LMICs with elevated TB incidence.

What do these findings mean?

- Introduction of a new TB vaccine was found to be impactful and cost-effective for a range of assumptions on vaccine price and delivery strategies, with aggregate health and economic benefits of similar scale to the most influential health interventions in LMIC settings in recent years.
- The results of these analyses can be used by global and country stakeholders to inform TB vaccine policy and introduction preparedness, as well as decision-making around future development, adoption, and implementation of novel TB vaccines.

4) Did your study have a prospective protocol or analysis plan? Please state this (either way) early in the Methods section.
   a. If a prospective analysis plan (from your funding proposal, IRB or other ethics committee submission, study protocol, or other planning document written before analyzing the data) was used in designing the study, please include the relevant prospectively written document with your revised manuscript as a Supporting Information file to be published alongside your study, and cite it in the Methods section. A legend for this file should be included at the end of your manuscript.
   b. If no such document exists, please make sure that the Methods section transparently describes when analyses were planned, and when/why any data-driven changes to analyses took place.
   c. In either case, changes in the analysis-- including those made in response to peer review comments-- should be identified as such in the Methods section of the paper, with rationale.

*Authors’ response:*

*We did not develop a prospective protocol or analysis plan for this work. It is typically rare for economic evaluations to have prospective protocols aside from evaluations that occur alongside clinical trials. We have added language regarding this to the beginning of the Methods section. Regarding changes to methodology due to data availability and calibration procedure for the epidemiological model, we have included more details in the appendix from Clark et al.*

**Methods, Page 8, Lines 23–29:**

We estimated a range of outcomes quantifying the health and economic impact of new vaccine introduction for affected countries. To do so, we used linked epidemiological and economic models to project changes in healthcare utilization, health outcomes, and healthcare costs for various vaccine introduction scenarios compared to a ‘no-new-vaccine’ counterfactual. (Full epidemiological model details have been previously reported by Clark and colleagues, and are briefly described in Appendix S1. Any changes to the analysis that were required are also described; no prospective analysis plan was developed.)

5) Please ensure that the study is reported according to the CHEERS 2022 checklist and include the completed checklist as Supporting Information. Please add the following statement, or similar, to the Methods: "This study is reported as per the Consolidated Health Economic Evaluation Reporting Standards 2022 (CHEERS 2022) statement (S1 Checklist)." Please access this link for more details on the checklist: https://bmcmedicine.biomedcentral.com/articles/10.1186/s12916-021-02204-0
   a. When completing the checklist, please use section and paragraph numbers, rather than page numbers.

*Authors’ response:*

*As requested, we have added a completed CHEERS 2022 checklist as Appendix S4, and have added the requested language to the cost-effectiveness analysis section of the Methods. We have referred to section and paragraph numbers, as specified. We will update the section for “Conflicts of interest” as appropriate given the removal of this information from the main manuscript per editor request (8).*

6) Please ensure you provide definitions for all abbreviations in your tables including USD

*Authors’ response:*

*We have added the definitions for the USD abbreviation to the table footnotes in the manuscript and the appendix.*

7) References:
   a. Please select the PLOS Medicine reference style in your citation manager. In-text reference call outs should be presented as follows noting the absence of spaces within the square brackets, e.g., “…population [1,2].”
   b. References should have no more and no less than six names before “et al.”
   c. Please ensure that weblinks are current and accessible to date.

*Authors’ response:*

*We have updated the reference style to be PLOS Medicine format, and confirmed all requests above are incorporated.*

8) Please remove the ‘Financial disclosure” and “competing interest,” statements from the main text. In the event of publication, this information will be published as metadata based on your responses to the submission form.

*Authors’ response:*

*We have removed this text as requested.*

Reviewer #1:

This study estimated the cost and cost-effectiveness, and net monetary benefit of new TB vaccine introduction, from health system and societal perspectives using the WHO Full Value of Vaccines Assessment framework. This research topic is of importance and the results of the study can inform global-level decision-making for novel TB vaccine investment and introduction. However, I think the method section of the study is not clearly and sufficiently described. Below are my specific comments.

1. Please provide line numbers for easier reference.

*Authors’ response:*

*We have added line numbers to the manuscript.*

2. Method, Analytic overview: How did you select the starting year of 2028? Why did you select 2028 instead of 2023 or 2030 as the starting year?

*Authors’ response:*

*Thank you very much for this question. The year 2028 was selected as the earliest country-specific introduction year to align with anticipated product availability following TB vaccine candidate trial completion and results, based on expert consultation and analysis.*

*This rationale is now described in the methods section, and also included in the appendix (Appendix S1, section S1.6, pages 10–13)*

**Methods, Page 9, Lines 51–53:**

We selected 2028 as the earliest country-specific introduction year to align with anticipated product availability following TB vaccine candidate trial completion.

3. Method, Vaccination scenarios, page 5: "we assumed an average ten-year duration of protection, with exponential waning." Do you have any reference/evidence for the assumption of ten-year protection and the exponential waning?

*Authors’ response:*

*Thank you for this question. From the WHO Preferred Product Characteristics for New Tuberculosis Vaccines, it is indicated that “ten years or more of protection should be conferred after primary immunisation,” which is why we considered an average ten-year duration of protection for the primary analyses. We also evaluated a scenario that assumes lifelong duration of protection. The shape of waning immunity was modelled as an exponential distribution based on similar shapes for waning vaccine immunity of BCG and other vaccines.*

*We have incorporated this information, and associated references, into the supplementary material in order to provide more details for readers (Appendix S1, section S1.4).*

**Appendix, Pages 8–9, Lines 83–98:**

The vaccine profile for an adult/adolescent vaccine and infant vaccine were based on the WHO Preferred Product Characteristics for New Tuberculosis vaccines [1]...

We assumed duration of protection was 10 years on average, in addition to a sensitivity analysis with lifelong duration of protection. The shape of waning immunity was modelled as an exponential distribution, based on similar shapes for waning vaccine immunity of BCG [2] and other vaccines [3, 4].

[1] World Health Organization. WHO Preferred Product Characteristics for New Tuberculosis Vaccines. Geneva: World Health Organization. Licence: CC BY-NC-SA 3.0 IGO. 13 July 2018. https://www.who.int/publications/i/item/WHO-IVB-18.06 (accessed 18 October 2021).
[2] Abubakar I, Pimpin L, Ariti C, Beynon R, Mangtani P, Sterne JA, et al. Systematic review and meta-analysis of the current evidence on the duration of protection by bacillus Calmette-Guérin vaccination against tuberculosis. Health Technol Assess. 2013;17(37):1-372, v-vi.
[3] Blackwood JC, Cummings DA, Broutin H, Iamsirithaworn S, Rohani P. Deciphering the impacts of vaccination and immunity on pertussis epidemiology in Thailand. Proc Natl Acad Sci U S A. 2013;110(23):9595-600.
[4] Lewnard JA, Grad YH. Vaccine waning and mumps re-emergence in the United States. Sci Transl Med. 2018;10(433).
4. Method, Vaccination scenarios, page 5: What is definition of the routine vaccination program? Do you assume the routine vaccination the same across all 105 LMICs? I think there may be huge variations between countries regarding their routine vaccination programs.

*Authors’ response:*

*For routine vaccination, we modelled a vaccination strategy characterized by delivery at fixed sites following a standard dosing schedule, allowing for consistent budgeting and allocation of health care workers. We did not model differences between individual country health systems and the strength of the routine vaccination program. We have added details to the methods, and included this as a limitation.*

**Methods, Page 6, Lines 43–45:**

We assumed the infant vaccine would be delivered through the routine vaccination program, with vaccine delivery at fixed sites following a standard dosing schedule.

**Discussion, Page 19, Lines 347–353:**

Finally, we did not consider all possible product and introduction scenarios, such as varying ages of vaccination, vaccine coverage targets, and scale-up trends by country, but demonstrated the potential value of novel TB vaccines according to specified characteristics. Future work developing detailed country-level models could take into account the health system capacity of each country and the underlying country-specific TB epidemiology by age to inform more realistic delivery scenarios.

5. Method, Vaccination scenarios, page 5-6: "the adolescent/adult vaccine delivered through routine vaccination of nine-year-olds plus a one-time vaccination campaign for ages 10+." Why did you choose a routine vaccination of nine-year-olds? Is a nine-year-olds routine vaccination common in most LMICs? Also, the name "adolescent/adult vaccine" seems a bit misleading given that a big part of the program is vaccination among children of 9 years old.

*Authors’ response:*

*Thank you for this question. The name of the adolescent/adult vaccine is consistent with the name used in the WHO Preferred Product Characteristics for New Tuberculosis Vaccines, which recommends the development of a novel vaccine suitable for adolescents and adults as well as a separate new vaccine for neonatal/infants.*

*The adolescent/adult vaccine was delivered routinely to those aged nine in our scenarios, aligning with previous TB vaccine modelling (Harris, et al. 2020) which assumed co-delivery of the novel TB vaccine with the human papillomavirus vaccine (HPV) in schools. The WHO recommends delivery of the HPV vaccine to ages 9–14 (WHO 2017). HPV vaccination at the lower end of the age range is anticipated in the long term, and therefore we selected age nine to receive the novel vaccine routinely. While it is likely that the optimal age for vaccination in each of the 105 countries may differ, we have chosen a consistent age group across countries to allow for easier comparison.*

*Future detailed country models will be able to evaluate the impact of variation in age targeting but for this paper we have included this as a limitation.*

**Discussion, Page 19, Lines 347–350:**

Finally, we did not consider all possible product and introduction scenarios, such as varying ages of vaccination, vaccine coverage targets, and scale-up trends by country, but demonstrated the potential value of novel TB vaccines according to specified characteristics.

6. Method, Vaccination scenarios, page 6: "stakeholders, we assumed a coverage target of 85% for the infant vaccine (average coverage of diphtheria-tetanus-pertussis third dose for LMICs11), 80% for routine delivery of adolescent/adult vaccine, and 70% of the adolescent/adult vaccination campaign." Assuming that all the 105 countries can achieve the same level of coverage with the same speed of reaching the target without considering the health system capacity of each country individually is highly unrealistic to me.

*Authors’ response:*

*Thank you for your question. The calculation of country-specific introduction years incorporates information on country immunization capacity and other related indicators, and in this way allows the timing of vaccination scale-up to change based on country-specific factors. We believe this allows for a more realistic representation of the overall pace of vaccine scale-up than considered by previous TB vaccine models.*

*For the final vaccination coverage achieved, we chose to have a common value across countries in order to have a single scenario that is easy to communicate, and then tested higher/lower values in sensitivity analyses. We agree with the reviewer that health system capacity will impact the final coverage level reached, unless there are deliberate efforts to strengthen TB vaccine roll-out in countries with weak existing systems. These factors are not considered in the analysis. It is difficult to represent all country-specific factors in a global analysis such as this, and have included the lack of country-specific target coverage and scale-up speed in the Limitations section. In the future, detailed country-level data and models could take into account country-specific scale-up speed and target coverage to address this point.*

**Discussion, Page 19, Lines 347–353:**

Finally, we did not consider all possible product and introduction scenarios, such as varying ages of vaccination, vaccine coverage targets, and scale-up trends by country, but demonstrated the potential value of novel TB vaccines according to specified characteristics. Future work developing detailed country-level models could take into account the health system capacity of each country and the underlying country-specific TB epidemiology by age to inform more realistic delivery scenarios.

7. Method, Vaccination scenarios, page 6: "We assumed country-specific introduction years from 2028-2047, determined based on indicators for disease burden, immunization capacity, classification of the country as an "early adopter/leader," lack of regulatory barriers, and commercial prioritization." How did you determine the introduction years based on this factors? I think the authors need to elaborate a bit more. Also, if you already considered the differences between countries regarding these factors, how could you still assume all countries will reach the same level of coverage with the same speed?

*Authors’ response:*

*Thank you for these questions and comments on the country-specific introduction years, as we believe that this aspect is a key novel contribution of this study, and previous modelling on this scale (Knight, et al. 2014) has not considered how vaccine introduction year would vary by country. We have now added a detailed description of how we derived these country-specific introduction years (Appendix S1, section S1.6, pages 10–13). This process included a consultation with experts from WHO, Gavi, PATH, PDVAC, CHAI, and industry partners to both identify factors influencing the likelihood of adoption timeline and weigh these factors according to prioritization of effect. We agree with the reviewer that in practice the speed of scale-up (following adoption) will also vary by country. We believe the differences in introduction year captures much of the variation that would be introduced by having the pace of scale-up vary by country, but agree this is a simplification and have listed this in the Limitations section (edited text shown under last comment).*

*As noted under the last bullet, we chose to have a common value across countries for final vaccination coverage in order to have a single scenario that is easy to communicate, and then tested higher/lower values in sensitivity analyses. In the future, detailed country-level data and models could take into account country-specific scale-up speed and target coverage to address this point (edited text shown under last comment).*

8. Method, Epidemiological outcomes and health service utilization, page 6: "…data and 10 due to unsuccessful calibration results." What are the criteria for unsuccessful calibration results? The authors need to elaborate.

*Authors’ response:*

*We determined that the calibration for a given country was unsuccessful if the calibration routine (history matching with emulation or the subsequent Approximate Bayesian Computation - Markov Chain Monte Carlo method) was unable to find a single parameter set that matched all calibration targets. We have now provided details in the appendix (Appendix S1, section S1.3, pages 5–7) on the epidemiological model calibration as well as which countries were excluded from the final list of calibrated countries.*

9. Method, Epidemiological outcomes and health service utilization, page 6: "In countries with a significant burden of HIV-associated TB.." what is the definition of countries with a significant burden of HIV-associated TB. You need to be more specific about it.

*Authors’ response:*

*The additional HIV structure was included if the proportion of tuberculosis cases among people living with HIV (PLHIV) was greater than or equal to 15%, and if HIV prevalence in the country was greater than 1%.*

*We have now specified this definition in the manuscript as well as providing further details in the appendix (Appendix S1, section S1.2, pages 1*–*5).*

**Methods, Pages 6–7, Lines 63–67:**

We analyzed the remaining 105 countries (Appendix S2), representing 93.3% of global TB burden. In countries in which the proportion of TB cases among people living with HIV was greater than or equal to 15%, and the HIV prevalence in the country was greater than 1%, the model included the effects of HIV and antiretroviral therapy (ART) on TB infection and progression risks (Appendix S1).

10. Method, Cost outcomes, page 7: "Diagnostics costs for drug-susceptible (DS) and rifampicin-resistant (RR) TB were obtained from published literature, and extrapolated to all LMICs by country income level." Do you mean you adjusted the average diagnostics costs from literature by each country's income level? If so, how did you do the adjustment? Need to elaborate.

*Authors’ response:*

*For diagnostics costs, we assigned the average cost by income level to each country in the associated income level grouping. We have revised the manuscript text to provide additional clarity. The edited text is shown below.*

**Methods, Pages 7–8, Lines 81– 83 and Lines 92–94:**

Diagnostics costs for drug-susceptible (DS) and rifampicin-resistant (RR) TB were obtained from published literature as average values for each country income level [1], which were assigned to each LMIC in the associated country income level grouping [2].

…The previous unit costs were inflated to 2020 values in local currency using the country consumer price index [3], and converted to 2020 US dollars using market exchange rates [4].

[1] Laurence YV, Griffiths UK, Vassall A. Costs to Health Services and the Patient of Treating Tuberculosis: A Systematic Literature Review. Pharmacoeconomics. 2015;33(9):939-55.
[2] World Bank. World Bank Country and Lending Groups. Washington, DC: The World Bank; 2019. <https://blogs.worldbank.org/opendata/new-country-classifications-income-level-2019-2020> (accessed 4 December 2020).
[3] International Monetary Fund. World Economic Outlook Database, October 2021. Last updated: 1 July 2021. [Online] Accessed 12 April 2022. Available at: <https://www.imf.org/en/Publications/WEO/weo-database/2021/October>.
[4] World Bank. World development indicators. Washington, DC: The World Bank; 2020. Last updated: 8 April 2022. [Online] Accessed 12 April 2022. Available at: <http://data.worldbank.org/>.
11. Method, Cost outcomes, page 7: "…we derived unit costs by extrapolating estimates reported by the Global Health Cost Consortium…" what did you mean by "extrapolating" and how did you do the extrapolation?

*Authors’ response:*

*We have added additional detail to the manuscript to describe the estimation of these unit costs. The edited text is shown below.*

**Methods, Pages 7–8, Lines 86–92:**

For ART costs, direct non-medical costs (travel, accommodation, food, nutritional supplements) to the patient, and productivity costs (income loss experienced by patients during TB care), we derived unit costs by extrapolating estimates reported by the Global Health Cost Consortium (sample size = 39) and WHO patient cost surveys (sample size = 20) with meta-regression models for the respective outcomes specified as generalized linear regression models (GLMs), assuming a Gamma distributed outcome, a log link function, and gross domestic product (GDP) per capita as the predictor.

12. Method, Cost-effectiveness analysis, page 8: "We also reported a specification in which costs are discounted but not health outcomes." I personally think that health outcomes should not be discounted. I would recommend making this the main assumption.

*Authors’ response:*

*We agree with the reviewer that it is important to consider cost-effectiveness analysis with both discounted and undiscounted health outcomes, and have included the undiscounted health outcomes scenario accordingly. For the main analysis we have retained the approach with discounting of both costs and health outcomes at the same rate, to allow comparability with other cost-effectiveness analyses (for which discounting of both outcomes and costs is conventional) and to follow published guidance for economic evaluation (such as the US Second Panel on Cost-Effectiveness in Health and Medicine, and the iDSI Reference Case for Economic Evaluation). As noted by the reviewer, we also report results for analyses in which health outcomes are not discounted. We have edited the Discussion section to highlight these undiscounted results:*

**Discussion, Page 17, Lines 297–301:**

Cost-effectiveness was stronger in analyses that discounted future costs but not future health outcomes. Final decisions about vaccine adoption will be made by local decision-makers, based on the values placed on health benefits, opportunity costs, the relative timing of health outcomes and costs, and other context-specific considerations.

13. Method, Statistical analysis, page 9: "We constructed probability distributions representing uncertainty in economic inputs and disability weights…" How did you construct the probability distributions? Did you assume normal distribution for those inputs? How did you determine the parameters of the distributions? I think the authors need to elaborate a bit more.

*Authors’ response:*

*We have added additional detail regarding the probability distributions and their definitions as the reviewer suggests.*

**Methods, Pages 9–10, Lines 129–136:**

We constructed probability distributions representing uncertainty in economic inputs and disability weights, specified as Gamma distributions for parameters defined over [0, ∞], and Beta distributions for parameters defined over [0, 1]. For each parameter the distribution mean was set equal to the point estimate, and the dispersion was set so an equal-tailed 95% interval reproduced the reported interval width. For parameters estimated from a meta-regression model (ART costs, patient costs) we simulated parameter values from each fitted regression model. We drew 1000 random values for each uncertain parameter.

Reviewer #2:

Thank you for the opportunity to review this comprehensive and well written analysis. It includes important information about the potential cost impact and cost-effectiveness of novel TB vaccines. It focuses on an improved infant vaccine (80% efficacy), and an adolescent/adult vaccine with 50% efficacy in both previously infected and uninfected persons. The methods and results are wide-ranging, covering 105 countries, and buttressed by many sensitivity and scenario analyses.

*Authors’ response:*

*We thank the reviewer for these kind words.*

Major comments:

1. The economic analysis builds on extensive epidemiologic modeling, the methods for and results of which are detailed in a separate manuscript available as a medRxiv preprint (and supporting supplements). While it would not be appropriate to repeat the same material in the economic analysis manuscript, some additional, reasonably high-level information about the epidemiologic modeling methods would be appropriate in the main text of the economic analysis manuscript, and especially in its supplement. For example, the figure showing the basic model structure for the dynamic epidemiologic model could be added to the supplement to the present manuscript. Ideally there would be sufficient information in the main text for readers to understand the broad outlines of the epidemiologic model, without having to refer elsewhere.

*Authors’ response:*

*Thank you very much for this suggestion. We agree that we would not want to repeat the same material to the same level of detail as the epidemiology manuscript, but that additional information regarding the epidemiologic methods would be appropriate and beneficial for readers.*

*To address this, we have updated the appendix (Appendix S1) to include the following items to help readers understand the epidemiologic model being used:*

- *The TB natural history model description and figure (section S1.1, page 1; Figure S1.1, page 3)*
- *The HIV structure description and figure (section S1.2, pages 1–2, Figure S2.1, page 4)*
- *An overview of the calibration methodology and reasons for exclusion from the final list of calibrated countries (section S1.3, pages 5–8)*
- *An overview of the vaccine profile (section S1.4, page 8) and vaccine delivery scenarios (section S1.5, pages 9–10)*
- *Methods for determining country specific introduction years (section S1.6, pages 10–13) and vaccine coverage targets (section S1.7, page 14)*

2. The major assumptions, parameters and values around key cost inputs for TB care need to be much better articulated. Again, I recognize that there may be extensive information in the references provided, but readers should not need to consult these for basic understanding. For example, it would seem appropriate to add to the supplement a table where key component cost inputs used for DS- and RR-TB care are provided for each of the 105 countries, including point estimates, ranges, and distributions (referred to in the first paragraph on p. 9, but not explained elsewhere). The same holds true for costs borne by patients and families. A high-level summary of these input health care and patient/family costs should also be provided in the main text, potentially grouped by country income group (e.g. low, low-middle, and high-middle income countries).

*Authors’ response:*

*Thank you for this suggestion. We have also added a new Table 1 providing a summary of these unit cost inputs by country income level. We have also added a citation to a manuscript that describes the estimation of the detailed country-level unit costs supporting this analysis. This manuscript is currently in the WHO clearance system, and a citation for the pre-print will be available and included in the current manuscript if it is accepted for publication.*

*We have added additional detail regarding the probability distributions assumed for the unit cost prediction models in the main manuscript text.*

**Methods, Pages 7–8, Lines 86–92:**

For ART costs, direct non-medical costs (travel, accommodation, food, nutritional supplements) to the patient, and productivity costs (income loss experienced by patients during TB care), we derived unit costs by extrapolating estimates reported by the Global Health Cost Consortium (sample size = 39) and WHO patient cost surveys (sample size = 20) with meta-regression models for the respective outcomes specified as generalized linear regression models (GLMs), assuming a Gamma distributed outcome, a log link function, and gross domestic product (GDP) per capita as the predictor.

**Methods, Pages 9–10, Lines 129–136:**

We constructed probability distributions representing uncertainty in economic inputs and disability weights, specified as Gamma distributions for parameters defined over [0, ∞], and Beta distributions for parameters defined over [0, 1]. For each parameter the distribution mean was set equal to the point estimate, and the dispersion was set so an equal-tailed 95% interval reproduced the reported interval width. For parameters estimated from a meta-regression model (ART costs, patient costs) we simulated parameter values from each fitted regression model. We drew 1000 random values for each uncertain parameter.

3. The authors should better defend/justify their primary focus on the 1x per-capita GDP per DALY averted threshold, as opposed to the alternatives they explore in sensitivity analysis. In addition, the "Best Buy $100" threshold is shown in the supplementary figures but not mentioned or defined in the main text.

*Authors’ response:*

*We have added additional detail regarding the selection and comparison of different thresholds to the Methods section, and described the factors around these decisions in the Limitations section.*

**Methods, Page 9, Lines 112–116:**

ICERs were compared to a range of country-specific cost-effectiveness thresholds to reflect the lack of consensus for a single threshold, including multiples of per-capita GDP (assuming 1x per-capita GDP as a proxy for willingness to pay in the base case), recent estimates of the opportunity cost of healthcare spending, and the WHO's universal “Best Buy” threshold of $100 per DALY averted.

**Discussion, Page 19, Lines 343–347:**

In our main analysis we used 1x per-capita GDP as a threshold to approximate willingness-to-pay per DALY averted, based on its broad historical utilization in economic evaluation studies. However, recent evidence suggests that a more stringent cost-effectiveness threshold may be appropriate [1, 2], in which case vaccination would be cost-effective in fewer countries, and estimates of return on investment would be lower.

[1] Ochalek J, Lomas J, Claxton K. Estimating health opportunity costs in low-income and middle-income countries: a novel approach and evidence from cross-country data. BMJ Glob Health. 2018;3(6):e000964.
[2] Woods B, Revill P, Sculpher M, Claxton K. Country-Level Cost-Effectiveness Thresholds: Initial Estimates and the Need for Further Research. Value Health. 2016;19(8):929-35.
4. Along similar lines, I am somewhat uncomfortable with the restriction of the incremental net monetary benefit analysis to countries where the ICERs fall below the relevant cost threshold per DALY averted. I think at minimum the authors should add to the supplement a couple of tables showing estimated iNMB for the 105 countries as a whole. Otherwise the analysis might be taken to imply that adoption of the vaccines should be limited to those countries where they appear cost-effective based on those somewhat arbitrary thresholds. I suspect this is not the authors' intent, and indeed in the discussion it would be relevant to discuss their use of such thresholds with a policy lens.

*Authors’ response:*

*We agree with the reviewer’s rationale, and we have added an additional column for all 105 countries to the current table for infant and adolescent/adult vaccines describing iNMB in the appendix. We have also added the following to the Discussion section regarding the use of alternative cost-effectiveness thresholds:*

**Discussion, Page 17, Lines 295–301:**

We estimated cost-effectiveness using a range of cost-effectiveness benchmarks, based on recent discussion of the validity of conventional standards [1-4]. Cost-effectiveness was stronger in analyses that discounted future costs but not future health outcomes. Final decisions about vaccine adoption will be made by local decision-makers, based on the values placed on health benefits, opportunity costs, the relative timing of health outcomes and costs, and other context-specific considerations.

[1] Woods B, Revill P, Sculpher M, Claxton K. Country-Level Cost-Effectiveness Thresholds: Initial Estimates and the Need for Further Research. Value Health. 2016;19(8):929-35.
[2] Loganathan T, Ng CW, Lee WS, Hutubessy RCW, Verguet S, Jit M. Thresholds for decision-making: informing the cost-effectiveness and affordability of rotavirus vaccines in Malaysia. Health Policy Plan. 2018;33(2):204-14.
[3] Thokala P, Ochalek J, Leech AA, Tong T. Cost-Effectiveness Thresholds: the Past, the Present and the Future. Pharmacoeconomics. 2018;36(5):509-22.
[4] Jit M. Informing Global Cost-Effectiveness Thresholds Using Country Investment Decisions: Human Papillomavirus Vaccine Introductions in 2006-2018. Value Health. 2021;24(1):61-6.
5. Perhaps my biggest concern about this manuscript relates to the novelty and impact of its key findings for policymakers and other stakeholders. Members of the author group are well aware of, reference (and in many cases contributed to) previous analyses that have addressed the potential epidemiologic and economic impact of novel TB vaccines. The authors need to more clearly articulate how their current findings fit in the context of previously published work in this area, including what is different/striking/more robust about their analysis and results—beyond the obvious fact that it covers a very large and diverse set of countries. The authors should also emphasize (at least in their discussion) the findings which they believe will further convince readers and stakeholders of the necessity and value of improved TB vaccines, beyond what is already known in this regard.

*Authors’ response:*

*Thank you for this suggestion. There are several factors that distinguish this analysis from the existing literature: firstly, the effort taken to construct a realistic vaccine adoption timeline, based on analysis of country past performance and stakeholder consultation. As a result, this analysis provides a more robust estimate of the potential timing of vaccine impact compared to past analyses, which is particularly important given the expected role of vaccines in contributing to the attainment of TB elimination targets for specific calendar years. Secondly, the examination of a range of scenarios for vaccine introduction. In particular, the comparison of the base-case scenario to an accelerated scale-up scenario demonstrates the additional health and economic gains that would be possible with more rapid vaccine adoption. Thirdly, the estimation of incremental net monetary benefit as a measure summarising the health and economic benefits of TB vaccine introduction. Combined with the large number of countries modelled, this analysis quantifies the overall global benefits of TB vaccination in a way that has not been done by prior analyses. This is particularly important for justifying the investments that still need to be made in vaccine development and preparation for deployment.*

*We have edited the discussion section to highlight these factors:*

**Discussion, Page 18, Lines 312–326:**

There are several factors that distinguish this analysis from past studies [1–4]: firstly, the steps taken to construct a realistic vaccine adoption timeline, based on an analysis of factors affecting country adoption decisions and stakeholder consultation. As a result, this analysis provides a more robust estimate of the potential timing of vaccine impact compared to past analyses, which is particularly important given the expected role of vaccines in contributing to the attainment of TB elimination targets for specific calendar years. Secondly, this study examined a wide range of scenarios for vaccine introduction, illustrating how the pace and extent of scale-up affect overall impact by 2050. In particular, the comparison of the base-case scenario to an accelerated scale-up scenario demonstrates the additional health and economic gains that would be possible with more rapid vaccine introduction. Thirdly, this study estimated incremental net monetary benefit as a single measure summarizing the health and economic benefits of TB vaccine introduction. Combined with the large number of countries modelled, this analysis quantifies the overall global benefits of TB vaccination in a way that can be set against the costs and other challenges that must be overcome to develop a new vaccine. This is particularly important for justifying the investments that still need to be made in vaccine development and preparation for deployment.

[1] Knight GM, Griffiths UK, Sumner T, Laurence YV, Gheorghe A, Vassall A, et al. Impact and cost-effectiveness of new tuberculosis vaccines in low- and middle-income countries. Proc Natl Acad Sci U S A. 2014;111(43):15520-5.
[2] Fu H, Lewnard JA, Frost I, Laxminarayan R, Arinaminpathy N. Modelling the global burden of drug-resistant tuberculosis avertable by a post-exposure vaccine. Nat Commun. 2021;12(1):424.
[3] Weerasuriya CK, Harris RC, McQuaid CF, Bozzani F, Ruan Y, Li R, et al. The epidemiologic impact and cost-effectiveness of new tuberculosis vaccines on multidrug-resistant tuberculosis in India and China. BMC Med. 2021;19(1):60.
[4] Bishai DM, Mercer D. Modeling the economic benefits of better TB vaccines. Int J Tuberc Lung Dis. 2001;5(11):984-93.

Minor comments:

1. It is not clear how this analysis, labeled as being conducted within the WHO Full Value of Vaccines Assessment Framework, differs methodologically from other analyses that simply considered both health system and societal perspectives.

*Authors’ response:*

*We thank the reviewer for this point, as this is an important issue to clarify. Our larger scope of work includes analyses focused on equity considerations and macroeconomic implications, but these analyses are forthcoming. We have revised the manuscript text to reflect that we are only reporting part of the WHO Full Value of Vaccines Assessment Framework in this analysis.*

**Background, Page 4, Lines 16–20:**

The WHO promotes the Full Value of Vaccines Assessment framework to improve decision-making around vaccine development and use. As part of a Full Value of Vaccines Assessment of novel TB vaccines, we estimated the costs, cost-effectiveness, and net monetary benefit of TB vaccine introduction, from health system and societal perspectives, to inform global-level decision-making for novel TB vaccine investment and introduction.

2. Results, p. 10: please specify more clearly what are undiscounted vs. discounted costs.

*Authors’ response:*

*We have added a clarification to the beginning of the Results section that we are referring to costs without a 3% annual discount rate when we say “undiscounted.”*

3. In the Supplement, please provide the appropriate citation and reference for the disability weights shown in table S2.

*Authors’ response:*

*We thank the reviewer for noting this oversight; we have added the appropriate citation.*

Reviewer #3:

Thank you for the opportunity to revise this great and important paper for the field.

*Authors’ response:*

*We thank the reviewer for his/her kind words on our manuscript.*

Find below some minor comments

Introduction. Initial sentence will be false in a matter of months. So, I would probably not rank the diseases in terms of mortality.

*Authors’ response:*

*The reviewer’s point is well noted. We have revised this sentence to be more general:*

**Background, Page 4, Line 2:**

Tuberculosis (TB) is one of the world’s leading causes of infectious disease death.

Methods. What was the scenario of a POI+POD vaccine in adults chosen and not consider them separately (given that it seems vaccines in pipeline are designed with the purpose of preventing either infection (which would indeed protect against disease) or just disease. In other words, in adults, you would likely need to administer POI+POD (two separate vaccines) and this would have cost implications. Of course, there might be POI candidates that work immunologically similarly as POD, but the effect against POD and POI might be different. In addition, I do not think this is how they are currently conceived.

*Authors’ response:*

*Thank you for inquiring about the prevention of infection (POI) or prevention of disease (POD) efficacy of our hypothetical vaccine. We based the vaccine characteristics on the WHO Preferred Product Characteristics for New Tuberculosis Vaccines. For a novel adolescent/adult vaccine, it is recommended that the new vaccine prevents active pulmonary TB disease.*

*Therefore, for the adolescent/adult vaccine in our scenarios, we modelled a single prevention of disease (POD) vaccine that would be efficacious if administered to both uninfected and infected individuals. We assumed that the vaccine would have no direct impact on the risk of infection.*

*We have added text in the manuscript to clarify that both the adolescent/adult and infant vaccines are prevention of disease vaccines, as well as providing additional detail in the appendix (section S1.4).*

**Methods, Page 5, Lines 37–42:**

…we evaluated two different vaccine product profiles: an infant ‘pre-infection’ prevention of disease vaccine (i.e., efficacious for individuals uninfected at time of vaccination) with 80% efficacy targeting neonates, and an adolescent/adult ‘pre- and post-infection’ prevention of disease vaccine (i.e., efficacious in all individuals aside from those with active TB disease at time of vaccination) with 50% efficacy, based on WHO Preferred Product Characteristics for New Tuberculosis Vaccines.

**Appendix S1, Pages 8–9, Lines 81–98:**

**S1.4. Vaccine profile**

The vaccine profile for an adult/adolescent vaccine and infant vaccine were based on the WHO Preferred Product Characteristics for New Tuberculosis vaccines [1], and are outlined in Table S3.1 below.

**Table S3.1.**	**WHO Preferred Product Characteristics for New Tuberculosis Vaccines.**

| **Vaccine** | **Host infection status at time of vaccination required for efficacy** | **Effect type** | **Vaccine efficacy** | **Duration of protection** |
| --- | --- | --- | --- | --- |
| Adolescent / Adult | Pre- and post-infection | Prevention of disease | 50% | Lifelong |
|  |  |  |  | 10 years |
| Infant | Pre-infection | Prevention of disease | 80% | Lifelong |
|  |  |  |  | 10 years |

Vaccine efficacy was assumed to be the same in both PLHIV and HIV-naïve recipients in countries incorporating the HIV structure, and in both younger age groups and older adults. The vaccine was assumed to have the same impact on preventing drug-susceptible and drug-resistant tuberculosis as specified in the WHO PPCs [1]. As we were modelling a prevention of disease vaccine, there was no direct impact on *Mtb* transmission or the force of infection.

We assumed duration of protection was 10 years on average, in addition to a sensitivity analysis with lifelong duration of protection. The shape of waning immunity was modelled as an exponential distribution, based on similar shapes for waning vaccine immunity of BCG [2] and other vaccines [3, 4].

[1] World Health Organization. WHO Preferred Product Characteristics for New Tuberculosis Vaccines. Geneva: World Health Organization. Licence: CC BY-NC-SA 3.0 IGO. 13 July 2018. https://www.who.int/publications/i/item/WHO-IVB-18.06 (accessed 18 October 2021).
[2] Abubakar I, Pimpin L, Ariti C, Beynon R, Mangtani P, Sterne JA, et al. Systematic review and meta-analysis of the current evidence on the duration of protection by bacillus Calmette-Guérin vaccination against tuberculosis. Health Technol Assess. 2013;17(37):1-372, v-vi.
[3] Blackwood JC, Cummings DA, Broutin H, Iamsirithaworn S, Rohani P. Deciphering the impacts of vaccination and immunity on pertussis epidemiology in Thailand. Proc Natl Acad Sci U S A. 2013;110(23):9595-600.
[4] Lewnard JA, Grad YH. Vaccine waning and mumps re-emergence in the United States. Sci Transl Med. 2018;10(433).

It would be great to know what "lack of critical calibration data or unsuccessful calibration results" means?

*Authors’ response:*

*Thank you for this question. Lack of critical calibration data included missing TB incidence, mortality, and/or case notification estimates, or missing demographic estimates, demographic projections, and/or contact matrices. An unsuccessful calibration result was deemed if we were unable to find 1000 parameter sets for the country that matched all targets using history matching with emulation or the subsequent Approximate Bayesian Computation - Markov Chain Monte Carlo method. The reasons for not matching the calibration targets include data inconsistencies and model misspecification leading to the inability of the model to fit targets simultaneously.*

*We have provided these details in the appendix (section S1.3) on the epidemiological model calibration methods as well as which countries were excluded from the final list of calibrated countries.*

**Appendix S1, Page 5–8, Lines 61–80:**

**S1.3.	Calibration methodology**

The model was fitted to epidemiologic calibration targets using history matching with emulation, implemented using the hmer R package [1, 2]. If countries were unable to find at least 1000 fully fitted parameter sets using this method, they were subsequently assessed using an Approximate Bayesian Computation using Markov Chain Monte Carlo method (ABC-MCMC). ABC-MCMC was conducted using the easyABC package in R, modified by Sebastian Funk, Gwenan Knight, and the Tuberculosis Modelling group at LSHTM for adaptive sampling and to accept seeded parameter values [1, 3]. We used parameter sets with the maximum number of targets fitted using history matching with emulation as a starting seed, with the ABC-MCMC algorithm continuously adapting using the last 1000 points and the noise factor set to 0.0001.

Analysis was performed on 105 countries from the 135 total low- and middle-income countries identified based on 2019 World Bank Income groups. There were 20 countries excluded from the initial calibration attempt due to missing crucial data required to attempt calibration, and 10 countries which were unable to be calibrated (could not find a parameter set that matched all targets using both history matching with emulation as well as ABC-MCMC). Reasons for exclusion from the final list of calibrated countries are provided in Table S2.1.

**Table S2.1.	Reasons for exclusion from the final list of calibrated countries.**

| **Country** | **Reason for Exclusion** |
| --- | --- |
| Algeria | Did not calibrate |
| American Samoa | Missing multiple critical epidemiological data for calibration, no contact matrices available |
| Belize | No case notification or incidence data for children |
| Bosnia and Herzegovina | Did not calibrate |
| Cabo Verde | Did not calibrate |
| Comoros | No case notification data |
| Democratic Republic of the Congo | No case notification data by age |
| Republic of the Congo | No population estimates |
| Democratic People's Republic of Korea | Missing multiple critical epidemiological data for calibration |
| Djibouti | No case notification data by age |
| Dominica | Missing multiple critical epidemiological data for calibration, no contact matrices available |
| Guinea-Bissau | Did not calibrate |
| Guyana | Did not calibrate |
| Federated States of Micronesia | Missing multiple critical epidemiological data for calibration, no contact matrices available |
| Grenada | Missing multiple critical epidemiological data for calibration, no contact matrices available |
| Haiti | Missing 2020 contact matrix |
| Jamaica | Did not calibrate |
| Kiribati | Missing 2020 contact matrix |
| Kosovo | Missing multiple critical epidemiological data for calibration |
| Lebanon | Missing 2020 contact matrix |
| Marshall Islands | Missing multiple critical epidemiological data for calibration, no contact matrices available |
| North Macedonia | Did not calibrate |
| Samoa | No case notification or incidence data for children |
| Somalia | No contact matrices available |
| St. Lucia | No case notification or incidence data for children |
| St. Vincent and the Grenadines | Did not calibrate |
| Tonga | Did not calibrate |
| Turkmenistan | Did not calibrate |
| Tuvalu | No contact matrices available |
| West Bank and Gaza | Missing multiple critical epidemiological data for calibration |

[1] Iskauskas A. hmer: History Matching and Emulation Package. R package. 17 May 2022. Available at: https://CRAN.R-project.org/package=hmer (accessed 12 July 2022).
[2] Scarponi D, Iskauskas A, Clark RA, Vernon I, McKinley TJ, Goldstein M, et al. Demonstrating Multi-Country Calibration of a Tuberculosis Model Using New History Matching and Emulation Package - Hmer. medRxiv; 2022. doi: https://doi.org/10.1101/2022.05.13.22275052.
[3] Jabot F, Faure T, Dumoulin N, Albert C. EasyABC: Efficient Approximate Bayesian Computation Sampling Schemes. R package. 2015. Available at: https://CRAN.R-project.org/package=EasyABC (accessed April 2022).

If risk of TB goes up in most countries after 15 years of age, why was the age of 9 y-old selected? If we assume the efficacy wanes (as you do), you probably want to vaccine very close to the age where the risk greatly increases.

*Authors’ response:*

*Thank you very much for this question and comment on the age of routine vaccination. The risk of developing TB disease is known to increase with age, but there is likely to be variability between countries surrounding the peak age and the optimal age of vaccination.*

*The adolescent/adult vaccine was delivered routinely to those aged nine in our scenarios, aligning with previous TB vaccine modelling which assumed co-delivery of the novel TB vaccine with the human papillomavirus vaccine (HPV) in schools. The WHO recommends delivery of the HPV vaccine to ages 9–14. Vaccination at the lower end of the age range is anticipated in the long term, and therefore we selected age nine to receive the novel vaccine routinely. In addition to the routine vaccine, we assume campaign delivery of the adolescent/adult vaccine to individuals ages ten and over to cover those aged older than nine.*

*While it is likely that the optimal age for vaccination in each of the 105 countries may differ, we have chosen a consistent age group across countries to allow for easier comparison. Future detailed country-level models will be able to evaluate the impact of variation in age targeting.*

*We have included this point as a limitation in the main text.*

**Discussion, Page 19, Lines 347–350:**

Finally, we did not consider all possible product and introduction scenarios, such as varying ages of vaccination, vaccine coverage targets, and scale-up trends by country, but demonstrated the potential value of novel TB vaccines according to specified characteristics.

Is there any booster to the initial vaccination in children? The text needs clarification.

*Authors’ response:*

*Thank you for this question. In this analysis we only assume initial vaccination with no booster, although future work could consider multiple doses and boosters.*

*We have added details to the appendix (section S1.5, pages 9–10) on the specifics of the vaccine scenarios to ensure that this is clear for readers. We have also added a two-dose vaccination scenario, additional details in subsequent comment.*

Should you specify in the text the HBC country in which infant vaccination would not be cost effective?

*Authors’ response:*

*In this analysis we deliberately focus on global results and results for major world regions, as we believe country-specific studies are needed to investigate the contextual factors relevant to individual countries. A global analysis such as this cannot consider all the factors that will matter for individual countries, and for this reason we do not want to highlight individual country results.*

What about considering in the sensitivity analysis several vaccine doses? I think that several doses might be required (as it happens with many vaccines). Otherwise, I would suggest to state clearly the analysis is based on a single shot and perhaps acknowledge so in the limitations.

*Authors’ response:*

*Thank you very much for this question. In this analysis we looked at administering one dose of the novel vaccine to align with the recommended schedule in the WHO PPCs which advises that “a minimal number of doses and boosters required” would be preferred. However, we agree that a two-dose vaccine course is a plausible scenario, and have added a scenario to examine a two-dose course of vaccination in addition to the base-case assumption.*

**Methods, Page 11, Lines 157–159:**

Compared to the base-case scenario assuming one vaccine dose, we estimated results assuming that two vaccine doses were required to achieve the same level of efficacy, i.e., a full vaccination course required 2x the base-case vaccine price of $4.60 and 2x the delivery cost.

**Results, Page 15, Lines 257–261:**

Assuming a two-dose vaccination course increased infant vaccination costs to $23.3 billion (Appendix S29) and adolescent/adult vaccination costs to $100 billion (Appendix S30). From the health system perspective, a two-dose vaccination course decreased the number of countries in which infant vaccination was cost-effective at a per-capita GDP threshold from 47 to 31 and in which adolescent/adult vaccination was cost-effective from 64 to 46 (Appendices S39–S40).

How do the costs and cost effectiveness compare to other vaccines (or similar models for other diseases, i.e malaria,hiv, pneumococcal disease ) Could you make a reasonable comparison?

*Authors’ response:*

*We have added a comparison regarding the general relationship between disease burden and cost-effectiveness, which is similar between this analysis and other published systematic reviews and meta-analyses of licensed vaccines.*

**Discussion, Page 17, Lines 292–294:**

This relationship of ICERs decreasing as disease burden increases has also been shown for several licensed vaccines, including HPV, malaria, and rotavirus [1-3].

[1] Janko MM, Joffe J, Michael D, Earl L, Rosettie KL, Sparks GW, et al. Cost-effectiveness of rotavirus vaccination in children under five years of age in 195 countries: A meta-regression analysis. Vaccine. 2022;40(28):3903-17.
[2] Rosettie KL, Joffe JN, Sparks GW, Aravkin A, Chen S, Compton K, et al. Cost-effectiveness of HPV vaccination in 195 countries: A meta-regression analysis. PLoS One. 2021;16(12):e0260808.
[3] Sauboin C, Van Bellinghen LA, Van De Velde N, Van Vlaenderen I. Economic Impact of Introducing the RTS,S Malaria Vaccine: Cost-Effectiveness and Budget Impact Analysis in 41 Countries. MDM Policy Pract. 2019;4(2):2381468319873324.
