# Supplementary material for: The cost and cost-effectiveness of novel tuberculosis vaccines in low- and middle-income countries: A modeling study
Source: PLoS Med. 2023 Jan 24;20(1):e1004155. doi: 10.1371/journal.pmed.1004155 (PMC9873163; doi:10.1371/journal.pmed.1004155)
Supplement: Attachment — Submitted filename: Response_to_Review_v2.docx [file pmed.1004155.s003.docx]

**Itemized Response for PMEDICINE-D-22-01725-R2**

Comments from the Academic Editor

1. COMPETING INTERESTS STATEMENT: You state in your financial disclosure that members of the funding body are also co-authors on the manuscript. Please update your COI statement to reflect this. Please include details of which co-authors (initials shall suffice) are members of the funding body and what each author’s role in the study was, within this statement

***Authors’ response:***

*The competing interests statement has been updated in the journal submission system to reflect the following text:*

*“Members of the funder (NG, MZ, SYS, RH, IGB, NN) participated as authors on the study and critically reviewed the analysis, reviewed and revised the manuscript, and approved the final manuscript as submitted. All other authors have declared that no competing interests exist.”*

2. AUTHOR SUMMARY: “tuberculosis disease” suggest avoiding use of the term “disease” perhaps “tuberculosis infection", or something similar - please check throughout and amend where necessary

***Authors’ response:***

*We have removed the phrase “TB disease” in the author summary and throughout the manuscript.*

3. FIGURES: Please ensure that colour schemes used are accessible to those with colour blindness (i.e. avoiding the use of green or red)

***Authors’ response:***

*We now use the ‘viridis’ and ‘RColorBrewer’ color packages for the palettes in all figures. The ‘viridis’ package was specifically developed to both ensure graphics are easier to read for those with color-blindness as well as print well in greyscale, and the ‘RColorBrewer’ package likewise includes an argument to ensure that color palettes displayed are color-blind friendly. This change has led to updated versions of Figure 2, Figure 3, and the appendix figures.*

*Additional information about these color palettes can be viewed here:*

[*https://cran.r-project.org/web/packages/viridis/vignettes/intro-to-viridis.html*](https://cran.r-project.org/web/packages/viridis/vignettes/intro-to-viridis.html)

[*https://rdrr.io/cran/RColorBrewer/man/ColorBrewer.html*](https://rdrr.io/cran/RColorBrewer/man/ColorBrewer.html)

4. DISCUSSION: Line 338: there is an additional line space separating the text, please correct

***Authors’ response:***

*This formatting error has been corrected.*

5. REFERENCES: Where more than one reference is cited, please remove the spaces separating the citations. For example, line 17: [5, 6] should read [5,6]

***Authors’ response:***

*We have made this formatting change throughout both the manuscript and the appendix.*

Reviewer #1

6. I am mostly satisfied with the responses and revisions made by the authors. The only concern I still have is the assumption that all 105 countries can achieve the same level of vaccine coverage with the same speed of reaching the target. There could be some simple way to account for the capacity of health system for each country. For example, the HAQ index produced by IHME might be used to account for different health system capacity in each country, which could potentially make the results more realistic and useful to policy makers from different countries.

***Authors’ response:***

*We thank the reviewer for this suggestion, and for their earlier comments calling attention to the impact of differences in health system capacity between countries. We agree that differences in health system capacity will matter, and edits made in response to prior comments are now reflected in the manuscript. We believe that future country-specific studies are the appropriate place to incorporate these factors, as well as allow iterative development of scenarios that reflect country conditions and policy preferences, as noted in the discussion section:*

*“Future work developing detailed country-level models could take into account the health system capacity of each country and the underlying country-specific TB epidemiology by age to inform more realistic delivery scenarios.”*

Reviewer #2

7. Thank you for your careful and extensive revisions to your manuscript, which I think have more than adequately addressed my and other reviewers' comments.

***Authors’ response:***

*We thank the reviewer for these kind words.*

8. I have two minor comments/suggestions: In your abstract, please clearly indicate that the modeled vaccines were for prevention of disease (i.e. not prevention of infection).

***Authors’ response:***

*We have revised ‘methods and findings’ section of the abstract to include the following:*

*“Vaccine scenarios considered two vaccine product profiles (one targeted at infants, one at adolescents/adults), both assumed to prevent progression to active TB.”*

9. It is a foregone conclusion that any analysis that discounts costs, but not health outcomes, will make any intervention appear "more" cost-effective than the corresponding analysis which discounts both. Hence I think you can remove general statements to this effect (e.g. p, 17, line 297). I know you are well aware of (and cite) the methodologic reasons for discounting both health outcomes and costs for purposes of CEA. There may be good reasons to report undiscounted health outcomes alone, and sometimes a fully undiscounted CEA as a sensitivity analysis (which can then be compared with the base case results). However, undue emphasis on an analysis which discounts only costs can be problematic. Certainly I think one should avoid making any substantive conclusions on this basis.

***Authors’ response:***

*We have removed the sentence indicated by the reviewer stating that cost-effectiveness was stronger when discounting costs but not health outcomes.*

Reviewer #3

10. Thank you for addressing my comments, well done.

***Authors’ response:***

*We thank the reviewer for his/her kind words on our manuscript.*
